# Exosome-Related FTCD Facilitates M1 Macrophage Polarization and Impacts the Prognosis of Hepatocellular Carcinoma

**DOI:** 10.3390/biom14010041

**Published:** 2023-12-28

**Authors:** Youyi Liu, Yifei Tang, Hongliang Jiang, Xiading Zhang, Xingyi Chen, Jingrou Guo, Cheng Jin, Minchen Wu

**Affiliations:** 1 Wuxi School of Medicine, Jiangnan University, Wuxi 214122, China; youyi@jiangnan.edu.cn (Y.L.); 6212805025@stu.jiangnan.edu.cn (Y.T.); jhl3216560542@163.com (H.J.); 6212805001@stu.jiangnan.edu.cn (X.C.); 6212805002@stu.jiangnan.edu.cn (J.G.); 2 Wuxi Higher Health Vocational Technology School, Wuxi 214000, China; zxiading@163.com; 3 Department of Hepatobiliary Surgery, Affiliated Hospital of Jiangnan University, Jiangnan University, Wuxi 214041, China

**Keywords:** hepatocellular carcinoma, bioinformatic analysis, exosome, FTCD, macrophage infiltration

## Abstract

Background: Exosomes are essential for hepatocellular carcinoma (HCC) progression and have garnered significant interest as novel targets for diagnostic, prognostic, and therapeutic approaches. This study aims to identify potential exosome-related biomarkers for the development of useful strategies for HCC diagnosis and therapy. Methods: Three datasets obtained from the Gene Expression Omnibus (GEO) were utilized to identify differentially expressed genes (DEGs) in HCC. Through Gene Ontology (GO) analysis and protein–protein interaction (PPI) network, overall survival (OS) analysis, Cox analyses, and diethylnitrosamine (DEN)-induced HCC mouse model detection, exosome-related hub gene was screened out, followed by a prognostic value assessment and immune-correlates analysis based on the Cancer Genome Atlas (TCGA) dataset. The hub gene-containing exosomes derived from Hepa1-6 cells were isolated and characterized using differential ultracentrifugation, transmission electron microscopy scanning, and Western blot. Ultrasound-guided intrahepatic injection, cell co-culture, CCK-8, and flow cytometry were performed to investigate the effects of the hub gene on macrophage infiltration and polarization in HCC. Results: A total of 83 DEGs enriched in the extracellular exosome term, among which, FTCD, HRA, and C8B showed the strongest association with the progression of HCC. FTCD was independently associated with a protective effect in HCC and selected as the hub gene. The presence of FTCD in exosomes was confirmed. FTCD-stimulated macrophages were polarized towards the M1 type and suppressed HCC cells proliferation. Conclusions: FTCD is a potential exosome-related biomarker for HCC diagnosis, prognosis, and treatment. The crosstalk between FTCD-containing exosomes and macrophages in HCC progression deserves further investigation.

## 1. Introduction

Hepatocellular carcinoma (HCC), accounting for 70% to 90% of liver cancer cases, is the third leading cause of cancer-related deaths [1,2]. Although surgical intervention has been the primary curative treatment for HCC, the prognosis for patients in advanced stages remains unfavorable. Unfortunately, only a small percentage of HCC patients (about 20~30%) are diagnosed at an early stage, with the majority of cases (>70%) being identified at an advanced stage, resulting in missed opportunities for optimal surgical intervention [3,4]. Given these circumstances, there is an urgent need to identify early diagnostic markers and therapeutic targets for HCC.

In recent years, there has been growing interest in the molecular pathogenesis of HCC, particularly in elucidating the interactions between tumor cells and the tumor microenvironment [5,6]. Within this complex network, exosomes have emerged as key mediators of intercellular communication [7]. Exosomes are small membrane vesicles containing various biomolecules such as lipids, proteins, DNAs, and various forms of RNA [8]. They play a critical role in cell-to-cell communication and have been implicated in tumor progression [9]. Studies have revealed that HCC-derived exosomes can modulate tumor immune evasion by up-regulating PD-L1 expression in tumor-associated macrophages through the STAT3 signaling pathway [10]. Exosomal circCCAR1 released by HCC cells contributes to immunosuppression and resistance against anti-PD1 immunotherapy by facilitating dysfunction in CD8^+^ T cells within the HCC microenvironment [11]. The unique phospholipid bilayer membrane structure of exosomes provides protection against degradation by endogenous enzymes, making them attractive candidates as potential diagnostic markers with significant research value. Several specific exosomal biomarkers have been identified in HCC [12,13]. For instance, circulating miR-92b shows promise as a potential biomarker for early prediction of posttransplant HCC recurrence, particularly in patients experiencing early recurrence of HCC [14]. Although research on exosomes and their involvement in HCC has made significant advancements, our understanding of the underlying mechanisms governing HCC formation remains fragmented and limited in depth. Further investigations are needed to unravel the intricate regulatory mechanisms by which exosomes contribute to HCC progression. Exploring these mechanisms will be crucial for the development of novel diagnostic and therapeutic strategies for HCC.

In this study, we aimed to identify the key exosome-related biomarkers during the progression of HCC, laying the foundation for subsequent functional investigations. Initially, three RNA-Seq datasets were screened from the NCBI Gene Expression Omnibus (GEO) database, including 359 HCC tumor tissues and their corresponding adjacent normal tissues, and the differentially expressed genes (DEGs) were analyzed. Subsequently, gene ontology (GO) terminology analysis, protein–protein interaction (PPI) network, and survival analysis were performed for the screening of exosome-associated hub genes. The expression levels of these hub genes were then measured in liver tissues obtained from diethylnitrosamine (DEN)-induced HCC mice and the control mice. Among these genes, only formimidoyltransferase-cyclodeaminase (FTCD) exhibited a remarkable degree of statistical significance within the multivariate Cox regression model and emerged as a potential exosome-related biomarker for early diagnosis and prognosis evaluation of HCC. Furthermore, the presence of FTCD in exosomes was confirmed. Interestingly, the correlation analysis based on the Cancer Genome Atlas (TCGA) dataset found that the expression of FTCD was significantly positively correlated with macrophage infiltration. A series of in vitro and in vivo experiments revealed that FTCD-stimulated macrophages were polarized towards the M1 type and suppressed HCC cells proliferation. Our findings provided possible potential targets for the diagnosis and treatment of HCC and established the grounds for further investigations into the HCC microenvironment.

## 2. Materials and Methods

### 2.1. Transcriptomic Analyses Data

Following a systematic review, three gene expression datasets (GSE121248, GSE36376, and GSE55092) were selected from the NCBI GEO database. All the datasets were RNA-seq data and performed as follows: total RNA extracted from corresponding liver tissue was amplified and subjected to biotin labeling, hybridization, staining, washing, and scanning procedures according to standard Affymetrix protocols. The RNA-Seq data from GSE121248 and GSE55092 were based on the GPL570 platform ([HG-U133_Plus_2] Affymetrix Human Genome U133 Plus 2.0 Array, Affymetrix, Santa Clara, CA, USA) and consisted of 70 HCC tumor tissues vs. 37 adjacent normal tissues and 49 HCC tumor tissues vs. 91 adjacent normal tissues, respectively [15,16]. The GSE36376 database, utilizing the GPL10558 platform (Illumina HumanHT-12 V4.0 expression beadchip, Illumina Inc., San Diego, CA, USA), included 239 HCC tumor tissues vs. 194 adjacent normal tissues [17]. All data are available online and free of charge. This study was approved by the Medical Ethics Committee of Jiangnan University (JNU20211217IRB02).

### 2.2. Identification and Enrichment Analysis of DEGs

The GEO2R online analysis tool was utilized to identify the DEGs between HCC tumor tissues and adjacent normal liver tissues. Upon accessing the relevant GEO data website, the built-in GEO2R link was selected, followed by clicking on “Define groups” for grouping configuration. Subsequently, all HCC tumor samples within the dataset were assigned to the experimental group, while all adjacent normal samples were designated as the control group. After keeping the other settings at their default configurations on the website, an online analysis was conducted, and all analysis results were exported in Excel format. In the dataset of the results, genes that met the cutoff criteria of an adjusted *p*-value < 0.05 and |logFC| > 1.0 were considered as DEGs. The DEGs from each dataset were exported as separate Excel files. To analyze the overlap of DEGs among the three datasets, a Venn diagram web tool was employed (https://bioinfogp.cnb.csic.es/tools/venny/, accessed on 21 November 2023). GO analysis contribute to the understanding of the molecular mechanisms underlying complex biological phenomena and can guide further experimental investigations. In this study, we utilized the database for annotation, visualization, and integrated discovery (DAVID) to visualize the enrichment of DEGs in terms of biological process (BP), cellular component (CC), and molecular function (MF) and pathways (with a False Discovery Rate value < 0.05).

### 2.3. PPI Network Construction, Survival Analysis, and Hub Gene Identification

The DEGs that showed enrichment in the “extracellular exosome” term were identified as the primary focus and subjected to analysis using the STRING database. PPI pairs with a combined score greater than 0.40 were extracted, and a PPI network was constructed using Cytoscape software (version 3.6.1). Subsequently, the CytoHubba plugin in Cytoscape was utilized to filter the hub genes based on the degree of each protein node, and the genes were ranked according to their degrees. In this study, the top ten genes with the highest degrees were considered for further analysis.

The relationship between the expression of genes and overall survival (OS) in HCC patients was analyzed using the TCGA dataset. RNA-sequencing expression profiles and corresponding clinical information for HCC were downloaded from the TCGA dataset. The data acquisition and application methods followed the guidelines and policies set forth by the dataset. Kaplan–Meier (KM) survival analysis with a log-rank test was performed to compare the differences in survival between groups with high and low gene expression. For the KM curves, *p*-values and hazard ratios (HR) with a 95% confidence interval (CI) were generated using log-rank tests and univariate Cox proportional hazards regression. A *p*-value of less than 0.05 was considered statistically significant. Results from above were comprehensively analyzed for the identification of hub gene.

### 2.4. Prognostic Value Assessment and Immune-Correlates Analysis

For a more comprehensive assessment of the prognostic value of hub gene, we analyzed the associations between the expression level and important clinical parameters, such as tumor grade and vascular invasion in HCC patients based on the TCGA and GEO dataset GSE20017 [18]. RNA-Seq data and corresponding clinical information, containing age, gender, TNM stages and tumor grade, were downloaded from the TCGA dataset. Subsequently, univariate and multivariate Cox regression analyses were computed using R statistical software (version 4.0.3). The forest was used to show the *p* value, HR and 95% CI of each variable through ‘forestplot’ R package. Variables with a *p* value less than 0.05 were considered statistically significant. Based on the results of multivariate Cox proportional hazards analysis, a nomogram was developed using the ‘rms’ R package, allowing for the prediction of the 1 to 5-year survival probabilities for HCC patients.

Furthermore, the data obtained from TCGA dataset were divided into two groups, named hub gene high-expression group and hub gene low-expression group. R software and EPIC algorithms were used to evaluate the immune score in the two groups, and the statistical difference of two groups was compared through the Wilcox test. The correlation analysis between hub gene expression and immune cells infiltration in HCC was also performed using R software, in which Spearman’s correlation analysis was used to describe the correlation between quantitative variables without a normal distribution. *p* values less than 0.05 were considered statistically significant. Additionally, to facilitate additional cellular experiments, the Single-Cell Atlas in Liver Cancer (https://scatlaslc.ccr.cancer.gov/) was used to analyze the primary cell types within liver cancer tissues that express hub gene [19,20].

### 2.5. Cell Culture, Supernatant Preparation and Transfection

The mouse HCC cell line Hepa1-6 (SCSP-512), mouse macrophages cell line Raw264.7 cells (SCSP-5036) and mouse normal liver cells AML12 (SCSP-550) were kindly provided by Stem Cell Bank, Chinese Academy of Sciences, located in Shanghai, China. The cells were authenticated by STR profiling according to the cell bank and passaged in the laboratory for fewer than 6 months after receipt. Hepa1-6 and Raw264.7 cells were cultured in high-glucose DMEM medium, and AML12 cells were cultured in DMEM/F-12 medium. The culture media was supplemented with 10% fetal bovine serum and 1% penicillin and streptomycin. The cells were maintained at 37 °C in a humidified atmosphere with 5% CO_2_ until they reached the logarithmic growth phase. For supernatant preparation, Hepa1-6 and AML12 cells were washed three times with sterile PBS and further cultured in exosome-free serum for an additional 48 h.

To perform transfection experiments, Hepa1-6 cells were transfected with the pcDNA3.1(-) vector and pcDNA3.1-FTCD plasmid using LipoFiterTM Liposomal Transfection Reagent from Hanbio (Shanghai, China), following the manufacturer’s instructions. The transfection efficiency was confirmed by Western blot. For FTCD small interfering RNA (siRNA) treatment with AML12 cells, FTCD siRNAs (si-FTCD) and control siRNA (si-NC) were purchased from Genechem (Shanghai, China) and performed according to the manufacturer’s instructions. The FTCD siRNA sequences targeted the coding region of the FTCD mRNA (Table S1). The best siRNA for inhibiting FTCD was screened by quantitative real-time PCR (qPCR).

### 2.6. Animal Experiments

All animal experiments were conducted in accordance with the NIH Guide for the Care and Use of Laboratory Animals. The experimental protocol involving animals was approved by the Animal Ethics Committee of Jiangnan University (JN. No20210630b1301201[205]).

For the construction of DEN-induced HCC mice model, two-week-old healthy wild-type (WT) male C57BL/6 mice, along with their mother, were obtained from Changzhou Cavens Laboratory Animal Co., Ltd. (Changzhou, China) and randomly assigned to two groups. The experimental group received intraperitoneal injections of N-Nitrosodiethylamine (DEN, Sigma, Saint Louis, MO, USA) normal saline solution at a dosage of 15 mg/kg once a day until weaning, followed by monthly injections of 50 mg/kg after weaning, for a duration of 7 months. The control group received an equivalent volume of normal saline solution (NS). Ultimately, a total of 27 mice (17 in the experimental group) survived until the end of the study. 

For the detection of the effect of FTCD on macrophage infiltration in vivo, the ultrasound-guided intrahepatic injection was performed using 18 six-week-old healthy wild-type (WT) male C57BL/6 mice as described previously [21]. Mice were imaged in B mode (2D) using a 40 MHz (RM-704) transducer with the Vevo 3100LT system (VisualSonics, Fujifilm, Bothell, WA, USA) to determine and visualize the largest liver lobe (Figure S1). A 1 mL Terumo Myjector 29-gauge insulin syringe (Terumo, Tokyo, Japan) was aligned and advanced into the liver by freehand using the ultrasound monitor for reference. A 50 µL bolus of 20,000 cells suspended in DMEM:Matrigel (1:1 *v*/*v*) was injected directly into the liver at a depth of 3 mm (Figure S1). The mice were randomly divided into three groups: normal control group (injected with DMEM only), pcDNA3.1 group (injected with Hepa1-6 cells stably transfected with pcDNA3.1 plasmid), and pcDNA3.1-FTCD group (injected with Hepa1-6 cells stably transfected with pcDNA3.1-FTCD plasmid), and group housed for 1 week.

### 2.7. Isolation and Characterization of Exosomes

In this study, exosomes were isolated from the supernatants of Hepa1-6 cells and AML12 using a differential ultracentrifugation method. The collected cell supernatant underwent a series of centrifugation steps to remove cellular debris and large vesicles. First, centrifugation was performed at 1000× *g* for 10 min at 4 °C to eliminate residual dead cells. Next, the supernatant was subjected to centrifugation at 3000× *g* for 15 min to remove cell debris, followed by centrifugation at 10,000× *g* for 30 min to further eliminate cell debris and large vesicles. The resulting supernatant was then filtered through a 0.22 μm filter and subjected to ultracentrifugation at 100,000× *g* for 70 min at 4 °C. The pelleted exosomes were washed and resuspended in a large volume of PBS. To recover the exosomes, another centrifugation step was performed at 100,000× *g* for 1 h at 4 °C. The obtained exosomes were either suspended in PBS for subsequent experiments or stored at −80 °C for long-term storage.

The morphology of the exosomes was verified using transmission electron microscopy. The size distribution and quantification of the exosome samples were analyzed using a nanoparticle tracking analyzer NS300 from Malvern Instruments (Malvern, UK). The results were expressed as the mean ± standard deviation (SD). The expression of exosome-specific markers, such as CD9 and TSG101, was confirmed by Western blot.

### 2.8. Quantitative Real Time-PCR and Western Blot Assay

Total RNAs were extracted from mouse liver samples using the Total RNA Extraction Reagent (Yeasen Biotechnology Co., Ltd. (Shanghai, China)). The cDNA synthesis was performed using the One-Step RT-PCR Kit from CoWin Biosciences. The mRNA expression levels of three potential biomarkers were quantified using SYBR Green-based real-time PCR (qPCR) with the qPCR SYBR Green Master Mix (Yeasen Biotechnology) and the LightCycler 480 II instrument (Roche, USA). The relative quantification of the target genes was determined using the 2^−ΔΔCt^ algorithm. The primer sequences for the target genes and the internal control gene are provided in Table S1.

The exosome suspension was lysed on ice using RIPA Lysis Buffer supplemented with protease inhibitors for 15 min. The lysates of exosomes were then subjected to SDS-PAGE electrophoresis to separate the proteins based on their molecular weight. Subsequently, the proteins were transferred from the gel to polyvinylidene difluoride (PVDF) membranes. The membranes were blocked using skim milk powder to prevent non-specific binding and incubated with primary antibodies overnight at 4 °C. The primary antibodies used in this study included anti-TSG101 (ab125011, diluted at 1:5000) from Abcam (Hong Kong, China), anti-CD9 (Cat No. 60232, diluted at 1:10,000), anti-FTCD (Cat No. 66979, diluted at 1:6000), anti-β-Actin (Cat No. 20536, diluted at 1:3000), anti-PKM2 (Cat No. 60286, diluted at 1:10,000), anti-PFKM (Cat No. 55628, diluted at 1:2000), and anti-HK2 (Cat No. 66974, diluted at 1:10,000) from Proteintech (Wuhan, China). After incubation with the primary antibodies, the membranes were washed and incubated with anti-rabbit secondary antibodies for 2 h at room temperature. Finally, the specific bands of the target proteins bound to the corresponding antibodies were visualized using an ECL detection kit (Nanjing, China), and imaged using a Tanon-5200Multi Imager (Tanon Life Science, Shanghai, China).

### 2.9. Cells Co-Culture and Cell Proliferation Assay

For cells co-culture, Hepa1-6 cells were transfected with the pcDNA3.1(-) plasmid or pcDNA3.1-FTCD plasmid. Appropriate numbers of transfected-Hepa1-6 cells and Raw264.7 cells were seeded into the upper and lower chambers of 0.4 μm Transwell plates, respectively. After being incubated for 48 h, the supernatant of the lower chamber was collected, mixed with an equal volume of complete medium, and used for the culture of Hepa1-6 cells.

For cell proliferation assay, Hepa1-6 cells were seeded into 96-well plates (1000 cells/well) and divide into three groups: normal control group (cultured with complete medium), pcDNA3.1 group, and pcDNA3.1-FTCD (respectively cultured with two kind of collected supernatant above). After incubation for certain times, the cells were added with 10 μL Cell Counting Kit 8 (Vazyme, Nanjing, China) and incubated at 37 °C and 5% CO_2_ for 4 h before measuring absorbance at 450 nm.

### 2.10. Flow Cytometry

For investigating the effect of FTCD on macrophage polarization in vitro, the supernatant of Hepa1-6 cells transfected with the pcDNA3.1(-) plasmid or pcDNA3.1-FTCD plasmid, as well as the supernatant of AML12 cells transfected with si-NC or si-FTCD was collected and used for the incubation of Raw264.7 cells for 48 h. Then, Raw264.7 cells were washed and resuspended with precooled PBS, and incubated with specific antibodies, PE-conjugated anti-mouse CD86 (11-0862-82, 0.125 μg/test) and FITC-conjugated anti-mouse CD206 (12-2061-82, 0.125 μg/test) from Thermo Fisher Scientific (Waltham, MA, USA), at 4 °C in the dark for 30 min. For detecting the effect of FTCD on macrophage infiltration in vivo, the liver was isolated from the intrahepatic injected mouse described above. Single-cell suspensions were made, and incubated with specific antibodies, including SB436-conjugated anti-mouse CD45 (62-0451-82, 0.5 μg/test), APC-conjugated anti-mouse F4/80 (17-801-82, 2 μg/test), PE-conjugated anti-mouse CD86 and FITC-conjugated anti-mouse CD206 from Thermo Fisher Scientific, at 4 °C in the dark for 30 min. Flow cytometry was performed according to standard procedures on a BD FACSArica III (Piscataway, NJ, USA).

### 2.11. Statistical Analysis

Each sample was subjected to three technical replicates in each trial. Statistical analysis was performed using SPSS 26.0 and GraphPad Prism 7 software. Data were presented as means ± SEM. Group differences were evaluated using Student’s *t*-test or the rank sum test, depending on the data distribution. Log-rank tests was used to compare the survival difference between two groups. Spearman’s correlation analysis was performed to describe the correlation between quantitative variables. The level of significance is indicated in the figures and figure legends as follows: no significance (ns), * *p* < 0.05, ** *p* < 0.01, *** *p* < 0.001, **** *p* < 0.0001.

## 3. Results

### 3.1. The DEGs Shared in Three HCC RNA-Seq Datasets Are Enriched in Extracellular Exosome

**Figure 1:**
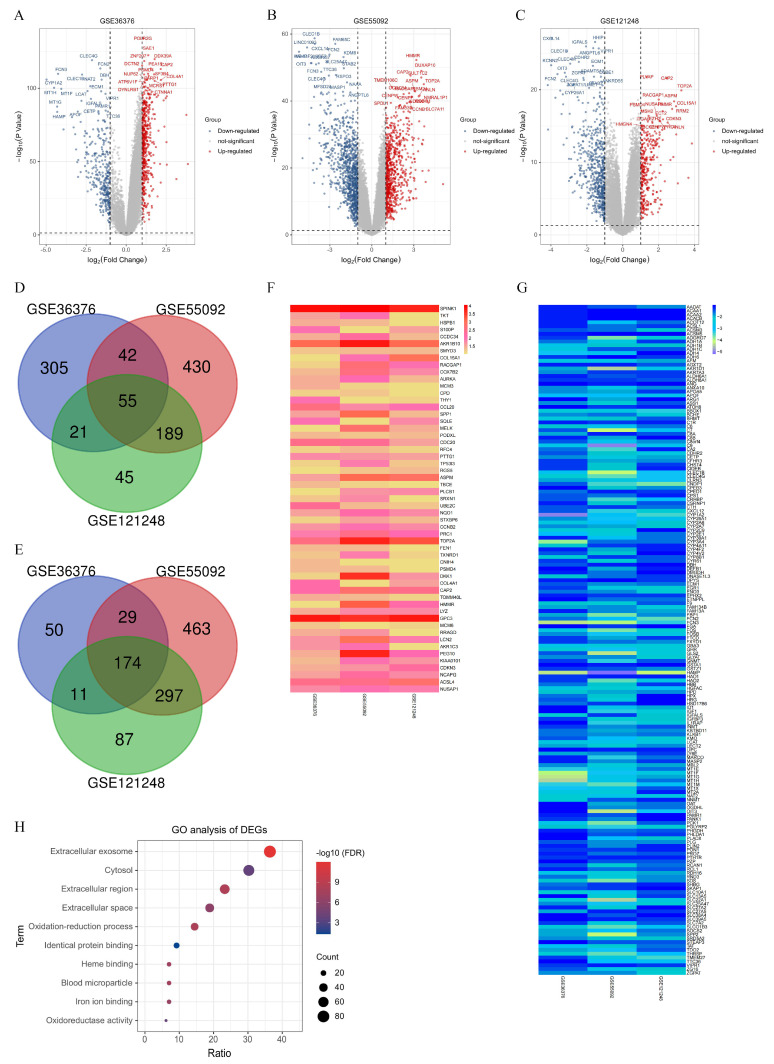
Analysis on the shared DEGs of GES36376, GSE55092, and GSE121248 dataset. (**A**) Volcano plot depicting the DEGs of GSE36376. (**B**) Volcano plot illustrating the DEGs of GSE55092. (**C**) Volcano plot showing the DEGs of GSE121248. (**D**) Venn diagram representing the shared up-regulated DEGs. (**E**) Venn diagram displaying the shared down-regulated DEGs. (**F**) Heatmap exhibiting the shared up-regulated DEGs. (**G**) Heatmap presenting the shared down-regulated DEGs. (**H**) The top ten GO analysis terms ranked by the number of gene counts. X-axis (Ratio) means the proportion of genes for the GO term relative to the total number of genes.

In this study, three gene expression datasets (GSE36376, GSE55092, and GSE121248) were analyzed to identify DEGs between HCC tumor tissues and adjacent normal liver tissues. From GSE36376 dataset, a total of 687 DEGs were identified, with 423 up-regulated genes and 264 down-regulated genes (Figure 1A). In GSE55092 dataset, 1679 DEGs were found, including 716 up-regulated and 963 down-regulated genes (Figure 1B). From GSE121248 dataset, we observed 879 DEGs, with 310 up-regulated genes and 569 down-regulated genes (Figure 1C). To explore the common DEGs across the datasets, a Venn analysis was performed. The results revealed that 229 DEGs were shared among the three datasets, with 55 up-regulated genes and 174 down-regulated genes (Figure 1D,E). The specific symbol names of the DEGs were displayed (Figure 1F,G, Table S2). The GO analysis was performed with a cutoff criterion of False Discovery Rate (FDR) value < 0.05 and gene counts ≥ 10. A total of 19 enriched GO terms were identified and classified into three groups (BP, CC and MF). The top 10 enriched GO terms are presented in Figure 1H. Notably, the term “extracellular exosome” was the most abundant term, accounting for approximately 36% of the shared DEGs.

### 3.2. Identification of Exosome-Related FTCD as a Hub Gene

**Figure 2:**
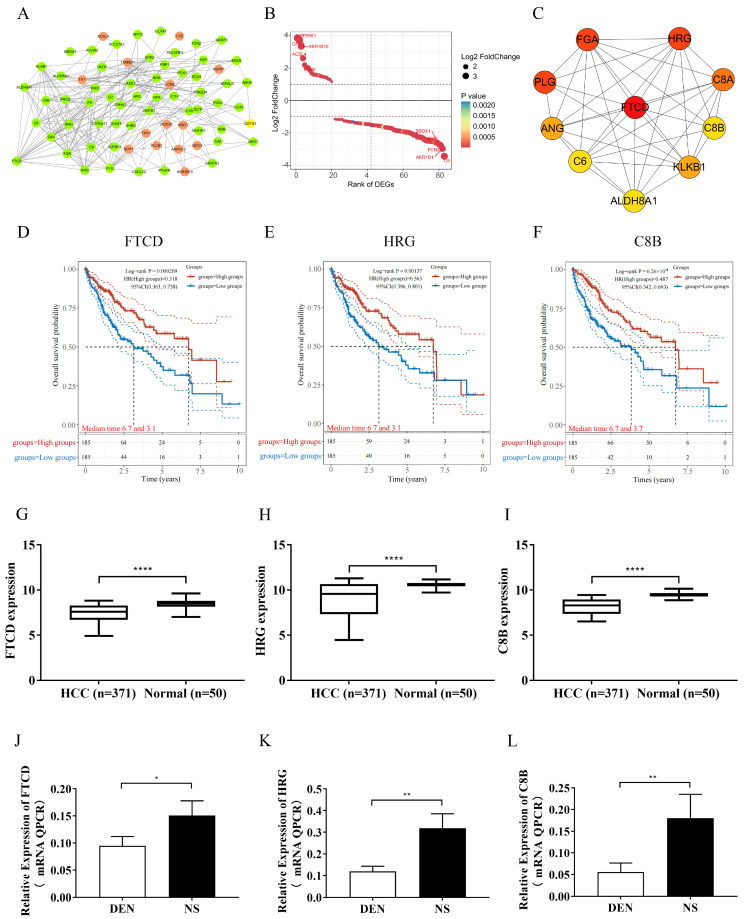
Identification of exosome-related hub gene. (**A**) The PPI network of exosome-related DEGs, the red dots represent up-regulated DEGs and green dots represent down-regulated DEGs. (**B**) Prioritization of exosome-related DEGs based on logFC. (**C**) Top 10 genes with high connectivity to surrounding genes. (**D**–**F**) Overall survival analysis of FTCD (**D**), HRG (**E**), and C8B (**F**). (**G**–**I**) Expression level of FTCD (**G**), HRG (**H**), and C8B (**I**) in HCC tissues and normal liver tissues based on TCGA dataset. (**J**–**L**) FTCD (**J**), HRG (**K**), and C8B (**L**) mRNA expression level in liver tissues of DEN-induced HCC mice model and control mice. Graphs represent means ± SEM from a minimum of three independent experiments. Log-rank tests were used to compare the survival difference between two groups. The statistical difference of the expression of genes between two groups was compared through the Wilcox test. * *p* < 0.05, ** *p* < 0.01, **** *p* < 0.0001.

To identify exosome-related hub genes and crucial gene modules involved in HCC progression, the STRING database and Cytoscape software (version 3.9.1) were utilized. From the STRING database, a total of 71 of the 83 exosome-related DEGs were filtered into the PPI network complex (Figure 2A,B, Tables S3 and S4). Next, the connectivity degree of the genes in the PPI network complex was calculated using the CytoHubba plug-in in Cytoscape software. The top ten genes with the highest scores were considered for further analysis. The top ten genes were FTCD (degree = 19), FGA (degree = 18), PLG (degree = 18), HRG (degree = 18), C8A (degree = 17), KLKB1 (degree = 15), ANG (degree = 15), C6 (degree = 14), ALDH8A1 (degree = 14), and C8B (degree = 14) (Figure 2C, Tables S5 and S6). 

The prognostic value of the top ten genes was investigated by analyzing the relationship between their mRNA expression levels and overall survival rate in HCC patients using data from TCGA dataset. Clinical data and mRNA expression data of 371 HCC tumor tissues and 50 normal liver tissues were obtained from the TCGA database. Survival data were available for 370 HCC cases. Among the ten genes, FTCD (HR = 0.518 [0.363–0.738], *p* = 0.000269), HRG (HR = 0.563 [0.396–0.801], *p* = 0.00137), and C8B (HR = 0.487 [0.342–0.693], *p* = 0.0000626) exhibited the top three best overall survival for HCC (Figure 2D–E and Figure S2, Table S7). The analysis of the TCGA dataset revealed that the mRNA expression levels of the three genes (FTCD, HRG, and C8B) were significantly down-regulated in HCC tissues compared to normal liver tissues (Figure 2G–I). To further validate the mRNA expression changes of the three genes in the pathogenesis of HCC, the mRNA expression levels of the genes were measured in liver tissues of DEN-induced HCC mice model using qPCR. The results showed that the mRNA expression levels of the three genes in DEN-induced HCC mice liver tissues were significantly lower than in the control mice liver tissues (Figure 2J–L). Taken together the above results, we identified FTCD as the exosome-related hub gene.

**Figure 3:**
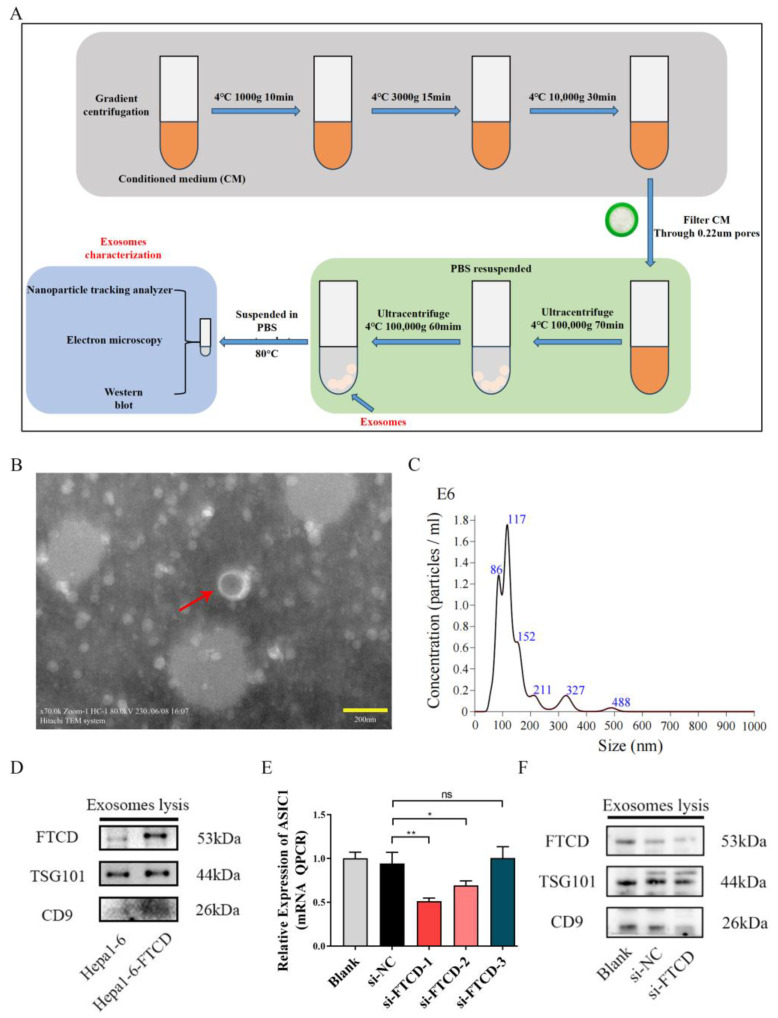
Extraction and identification of exosomes. (**A**) Flow chart for exosome extraction. (**B**) Transmission electron microscopy images of exosomes. Scale bar, 200 nm. (**C**) The particle size distribution of extracted exosomes by nanoparticle tracking analysis. (**D**) The presence of FTCD and exosome marker proteins (TSG101 and CD9) in Hepa1-6-derived exosomes detected by Western blot. (**E**) Evaluation of FTCD knockdown efficacy of three different siRNA using qPCR. Graphs represent means ± SEM from a minimum of three independent experiments. (**F**) The presence of FTCD and exosome marker proteins (TSG101 and CD9) in AML12-derived exosomes detected by Western blot. Statistical significance was analyzed by two-tailed *t*-test. * *p* < 0.05, ** *p* < 0.01.

An online platform (https://scatlaslc.ccr.cancer.gov/) was employed to analyze the primary cell types within liver cancer tissue that express FTCD. The results indicated that, among various cell types within liver cancer tissues, FTCD was predominantly expressed in hepatocytes and liver malignant cells, rather than in immune cells such as T cells (Figure S3). To further validate the expression of FTCD in exosomes, exosomes were isolated and characterized from the cell supernatants of Hepa1-6 and Hepa1-6 cells overexpressing FTCD. A detailed description of experimental and material parameters was given in the Section 2 (Figure 3A). Transmission electron microscopy analysis confirmed the presence of cup-shaped morphology in the exosome particles (Figure 3B). Nanoparticle-tracking analysis demonstrated an average particle size of 138.8 nm for the isolated exosomes (Figure 3C). Western blot assay was performed, targeting two commonly used exosome marker proteins (TSG101 and CD9) and FTCD. The results demonstrated that FTCD was indeed present in exosomes and can be secreted through exosomes (Figure 3D and Figure S4). Furthermore, after siRNA transfection, qPCR results indicated that si-FTCD-1 exhibited the most pronounced downregulation effect on FTCD expression levels (Figure 3E). Following si-FTCD-1 transfection into AML12 cells, exosomes were isolated and analyzed. The findings revealed that the FTCD levels within exosomes from normal AML12 cells were higher than those from cells with downregulated FTCD levels (Figure 3F and Figure S4).

### 3.3. High Expression of FTCD Is Associated with Macrophage Infiltration and a Favorable Prognosis in HCC

**Figure 4:**
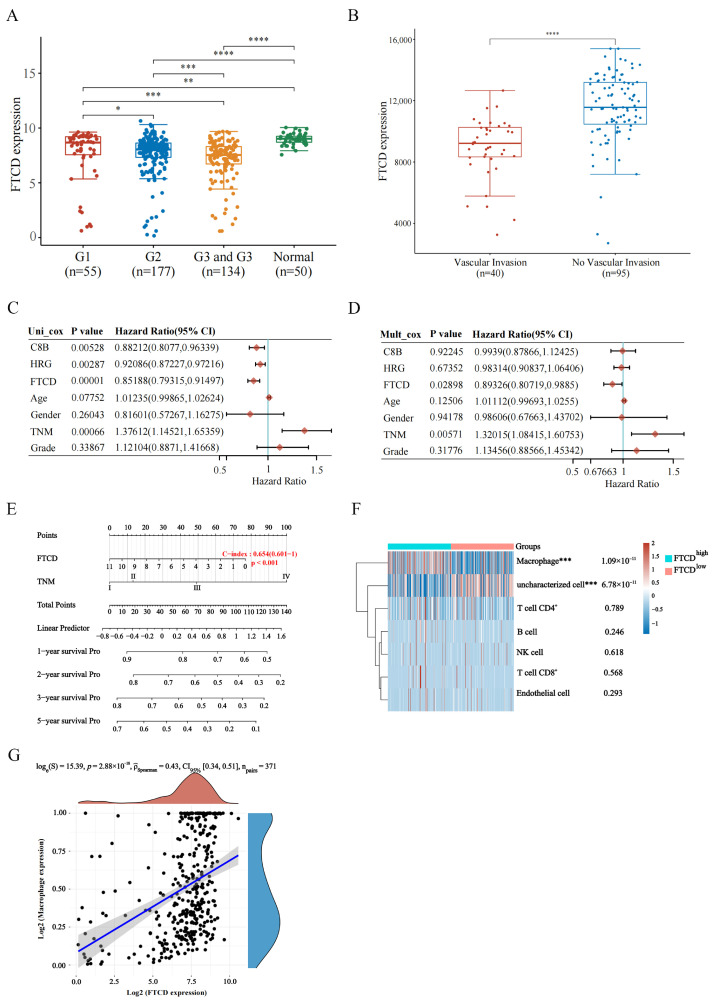
Prognosis and immune correlation analysis of FTCD based on TCGA dataset and GEO dataset. (**A**) The mRNA expression level of FTCD in HCC patients with different grades based on TCGA dataset. (**B**) The mRNA expression level of FTCD in patients with or without vascular invasion using GEO20017 dataset. (**C**,**D**) Univariate Cox analysis (**C**) and multivariate Cox regression analysis (**D**) of potential biomarkers and other risk factors for HCC based on TCGA dataset. (**E**) Nomogram depicting the combined predictive ability of FTCD and TNM stage for 1 to 5-year overall survival prediction in HCC patients based on TCGA dataset. (**F**) Immune cell score heatmap based on TCGA dataset, different colors represent different expression distribution in FTCD high-expressed HCC tissues (FTCD^high^) and FTCD low-expressed HCC tissues (FTCD^low^). (**G**) The correlation between FTCD expression and macrophage expression. The statistical difference of two groups was compared through the Wilcox test. Spearman’s correlation analysis was used to describe the correlation between quantitative variables. * *p* < 0.05, ** *p* < 0.01, *** *p* < 0.001, **** *p* < 0.0001.

To further explore the prognostic value of FTCD, the relationships between FTCD expression and HCC clinical phenotypes, including tumor grade and vascular invasion, were analyzed using the TCGA dataset and GEO dataset. Among the 371 HCC samples in TCGA dataset, 366 had available tumor pathology data (Table S7), including 55 cases of well-differentiated tumors (grade 1, G1), 177 cases of moderately differentiated tumors (grade 2, G2), and 134 cases of poorly differentiated and undifferentiated tumors (grade 3 and grade 4, G3 and G4). The results showed that the later the grade of HCC, the lower the expression of FTCD (Figure 4A). In GEO dataset GSE20017, containing 40 HCC cases with vascular invasion and 95 cases without vascular invasion, FTCD was expressed at a higher level in HCC tissues without vascular invasion than in HCC tissues with vascular invasion (Figure 4B). The results of univariate and multivariate Cox regression analysis revealed that the high expression level of FTCD was an independent protective factor for prognosis in patients with HCC (Figure 4C,D). Based on the results of multivariate Cox proportional hazards analysis, a nomogram was developed incorporating FTCD and TNM stage to predict the 1 to 5-year survival probability (Figure 4E). Furthermore, the correlation analysis between FTCD expression and infiltrating immune cells in HCC was performed, demonstrating that FTCD expression was significantly positively correlated with macrophage infiltration (Figure 4F,G).

### 3.4. FTCD-Stimulated Macrophages Are Polarized towards the M1 Type and Suppress HCC Cells Proliferation

**Figure 5:**
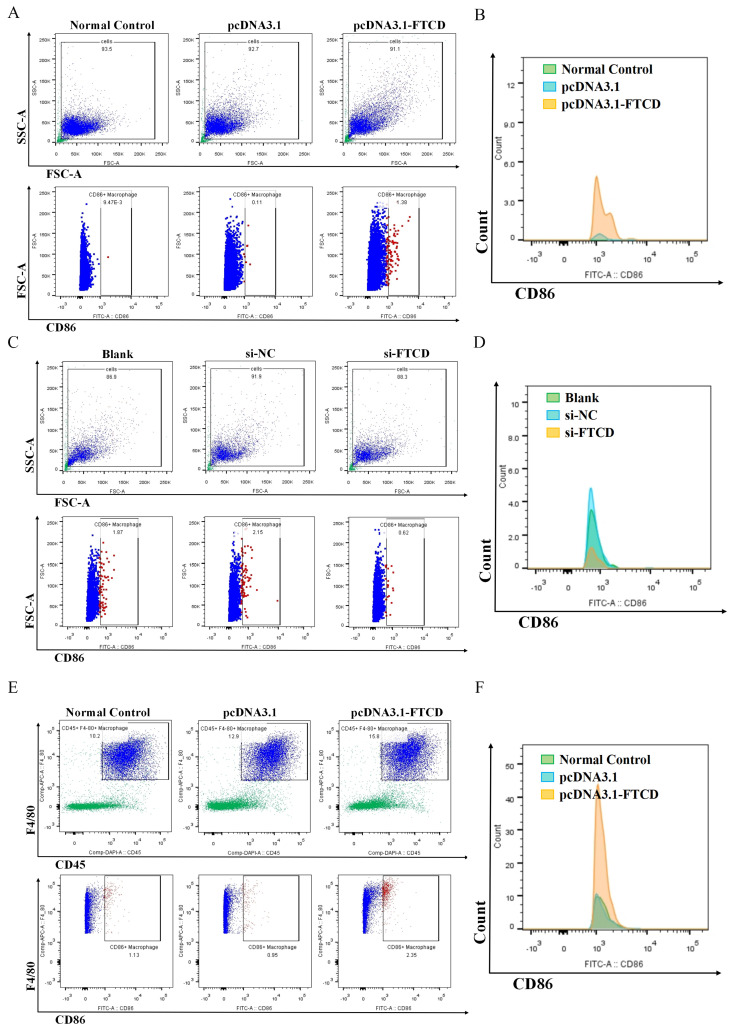
FTCD-stimulated macrophages were polarized towards the M1 type. (**A**,**B**) The rate of M1 polarization, as well as the expression of M1 marker CD86, was detected in Raw264.7 cells stimulated with FTCD overexpressed Hepa1-6-derived exosomes and control groups using flow cytometry. (**C**,**D**) The rate of M1 polarization, as well as the expression of M1 marker CD86, was detected in Raw264.7 cells stimulated with FTCD downregulated AML12-derived exosomes and control groups using flow cytometry. (**E**,**F**) The rate of M1 infiltration, as well as the expression of M1 marker CD86, was detected in livers of ultrasound-guided intrahepatic injection mice using flow cytometry.

**Figure 6:**
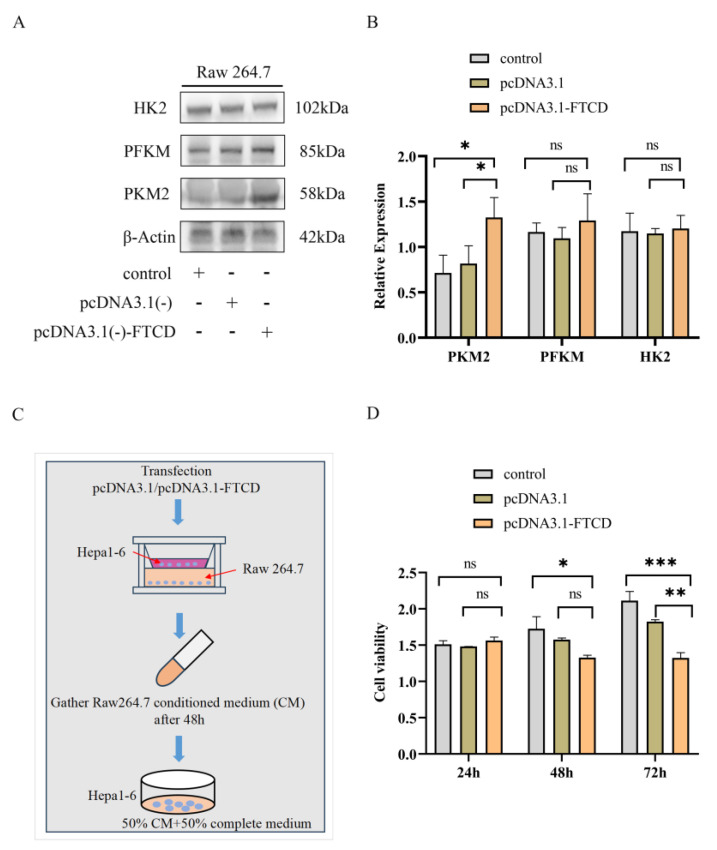
FTCD-stimulated macrophages exhibited PKM2 upregulation and suppressed HCC cells proliferation. (**A**,**B**) The expression levels of key enzymes (PKM2, PFKM, HK2) in glycolysis progression were detected in stimulated Raw264.7 using Western blot. (**C**) Flow chart for the culture of Hep1-6 cells using the supernatants collected from co-cultured Raw264.7 cells and FTCD-upregulated Hepa1-6 cells. (**D**) Cell proliferation of Hep1-6 cells cultured by the supernatants collected from co-cultured Raw264.7 cells and FTCD-differently-expressed-Hepa1-6 cells using CCK-8 assay. All graphs show the means ± SEM of at least three independent experiments. Statistical significance was analyzed by two-tailed *t*-test. * *p* < 0.05, ** *p* < 0.01, *** *p* < 0.001.

To probe the effects of FTCD on macrophage infiltration and polarization in HCC, a series of in vitro and in vivo experiments were performed as described in the Section 2. The results of flow cytometry suggested that the overexpression of FTCD in Hepa1-6 cells could promote M1 macrophage polarization in vitro (Figure 5A,B) and increase M1 macrophage infiltration in vivo (Figure 5E,F). While, after downregulating the expression of FTCD, the stimulatory effect of AML12 cell-derived exosomes on M1 macrophage polarization is attenuated (Figure 5C,D). Given that macrophage phenotypes were linked to their metabolic features and M1 macrophages were characterized by predominantly glycolytic metabolism [22,23], the protein expression of key enzymes in glycolysis progression was detected in stimulated Raw264.7 by Western blot. The results revealed that the overexpression of FTCD in Hepa1-6 cells could have up-regulated the expression of PKM2 in Raw264.7 (Figure 6A,B and Figure S5). Then, the supernatants from co-cultured Raw264.7 cells and FTCD-upregulated Hepa1-6 cells were collected and used for the stimulation of Hepa1-6 cells. The results of CCK-8 assay indicated that supernatants of FTCD-stimulated Raw264.7 cells could inhibit the proliferation of Hepa1-6 cells (Figure 6C,D).

## 4. Discussion

In recent years, the advancement of proteomics technology has allowed for a better understanding of the composition and function of proteins within exosomes [24]. Tumor cells are known to produce a significantly higher amount of exosomes compared to other components within the tumor microenvironment [25]. Proteins carried by exosomes exhibit differential expression patterns across different tumor types or stages [8]. These differences reflect the selective packaging and release of specific proteins by tumor cells, highlighting the functional significance of exosomes in intercellular communication and tumor microenvironment modulation and providing valuable insights into tumor progression, metastasis, and therapeutic response [26]. The study of exosomal proteins has opened up new avenues for understanding the molecular mechanisms underlying tumorigenesis and has the potential to revolutionize cancer diagnosis, treatment, and management [27].

In this study, a comprehensive analysis of three gene expression datasets (GSE121248, GSE36376, and GSE55092) containing HCC tumor tissues and adjacent normal tissues from the GEO database was conducted. Ten hub genes associated with exosomes (FTCD, FGA, PLG, HRG, C8A, KLKB1, ANG, C6, ALDH8A1, and C8B) were screened out, among which FTCD was emerged as the key potential exosome-related biomarker having potential value for the diagnosis and prognosis of HCC. FTCD is expressed in almost all mammalian organs, with the liver showing the highest expression level [28]. Recent literature suggests that FTCD not only possesses enzyme function but also plays a structural role in connecting biofilms. Additionally, FTCD has been proposed as a potential tumor suppressor gene for HCC [29,30]. Previous studies investigating the relationship between FTCD and HCC have revealed that FTCD is a downstream target of HIF-1α, which serves as an unfavorable prognostic indicator for HCC. HIF-1α regulates the occurrence and progression of HCC through the PI3K/AKT pathway [31,32]. Our findings support the notion that FTCD, as the key potential exosome-related biomarker in HCC, can be detected in human urinary exosomes [33]. Moreover, FTCD is targeted by anti-liver cytosol type 1 antibodies, which are characteristic of type 2 Autoimmune Hepatitis [34]. Excessive and inappropriate autoimmunity is a primary cause of AIH, while the decrease in anti-tumor immunity is closely associated with HCC development [35]. Furthermore, emerging research indicates that HCC-derived exosomes can suppress the cytotoxicity of T-cells and Natural Killer cells, promote the immunosuppressive M2 macrophages, and facilitate immune evasion during HCC development [36]. These highlight the importance of investigating the interplay between FTCD, exosomes, and tumor immunity, as it may provide valuable insights for HCC diagnosis and treatment strategies.

In the present study, we analyzed the correlation of FTCD expression with immune cell infiltration based on the TCGA dataset and found that FTCD exhibited a significant positive association with the infiltration of macrophages. The in vitro and in vivo experiments revealed that FTCD-stimulated macrophages were polarized towards the M1 type and could have suppressed the proliferation of HCC cells. Macrophages, as innate immune cells, exhibit high plasticity and heterogeneity within the immune system. They can be activated into two distinct pathways, classical pathway M1 and alternative activation M2, in response to various microenvironmental stimuli [37]. Numerous studies have demonstrated the involvement of macrophages in various aspects of tumor development, including angiogenesis, invasion, and migration within the tumor microenvironment [38]. In particular, M2-type macrophage contributes to HCC malignant progression and promotes immune escape [39]. It is worth noting that different types of exosomes have been discovered to play a role in regulating macrophage polarization. Tumor cell-derived exosomes promote the immunosuppressive polarization of macrophages by mediating glycolytic metabolic reprogramming, which leads to tumor metastasis [40]. For instance, HCC-derived exosomal circTMEM181 promotes immunosuppression by targeting the expression of CD39 in macrophages and improves the drug resistance of patients with anti-PD-1 therapy [41]. In view of these, our findings are worthwhile for further investigation of the crosstalk between FTCD-containing exosomes and various cellular components within the HCC immune microenvironment.

Experimental approaches such as co-culture systems, flow cytometry, and immune cell functional assays can be employed to study the impact of FTCD-containing exosomes on immune cell activation, differentiation, and immune response modulation. Additionally, studying the molecular mediators and signaling pathways involved in the crosstalk between FTCD-containing exosomes and immune cells can help uncover the mechanisms. These studies can provide valuable insights into the complex interplay between tumor cells, exosomes, and immune cells, and potentially lead to the development of novel therapeutic strategies targeting FTCD and exosome-mediated pathways, as well as inform the development of immunotherapeutic approaches for HCC treatment.

In conclusion, through bioinformatic analysis of RNA-Seq datasets and gene expression detection of the DEN-induced HCC mouse model, exosome-related FTCD was identified as a potential diagnostic and prognostic biomarker in HCC. Further cell and animal experiments revealed that FTCD-stimulated macrophages were polarized towards the M1 type and suppressed HCC cells proliferation. Further comprehensive investigations are necessary to validate these findings before considering FTCD as a new drug target and prognostic indicator in clinical practice for HCC.

## 5. Conclusions

FTCD is a potential exosome-related biomarker for HCC diagnosis, prognosis, and treatment. The crosstalk between FTCD-containing exosomes and macrophages in HCC progression deserves further investigation.

## Data Availability

Datasets in this work are from public database. All data generated during this study are included in the manuscript and Supplementary Information.

## Supplementary Materials

The following supporting information can be downloaded at: https://www.mdpi.com/article/10.3390/biom14010041/s1, Figure S1: Ultrasound-guided intrahepatic injection; Figure S2: Overall survival analysis. (A) C6. (B) C8A. (C) PLG. (D) ALDH8A1. (E) FGA. (F) ANG. (G) KLKB1. *p* < 0.05 was considered as statistically significant; Figure S3: The expression of FTCD in different cell types within liver cancer tissues; Figure S4: The original image of the exosomes detection using Western blot; Figure S5: The original image of the detection of the expression of key enzymes in glycolysis progression using Western blot; Table S1: Primers and siRNAs used in the study; Table S2: The DEGs shared in GSE36376, GSE55092, and GSE121248 datasets; Table S3: The 83 exosome-related DEGs; Table S4: PPI network information of the exosome-related DEGs; Table S5; Hub genes with highest degree of connectivity; Table S6: PPI network information of the top 10 genes; Table S7: The clinical background data and gene expression value of HCC obtained from TCGA dataset.
