# Supplementary material for: Exosome-Related FTCD Facilitates M1 Macrophage Polarization and Impacts the Prognosis of Hepatocellular Carcinoma
Source: Biomolecules. 2023 Dec 28;14(1):41. doi: 10.3390/biom14010041 (PMC10813691; doi:10.3390/biom14010041)

Figure S1 Ultrasound-guided intrahepatic injection.

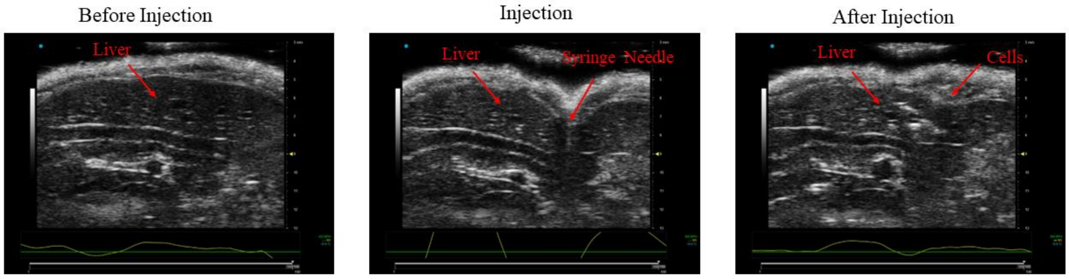

Figure S2 Overall survival analysis. (A) C6. (B) C8A. (C) PLG. (D) ALDH8A1. (E) FGA. (F) ANG. (G) KLKB1. $P < 0.05$ was considered as statistically significant.

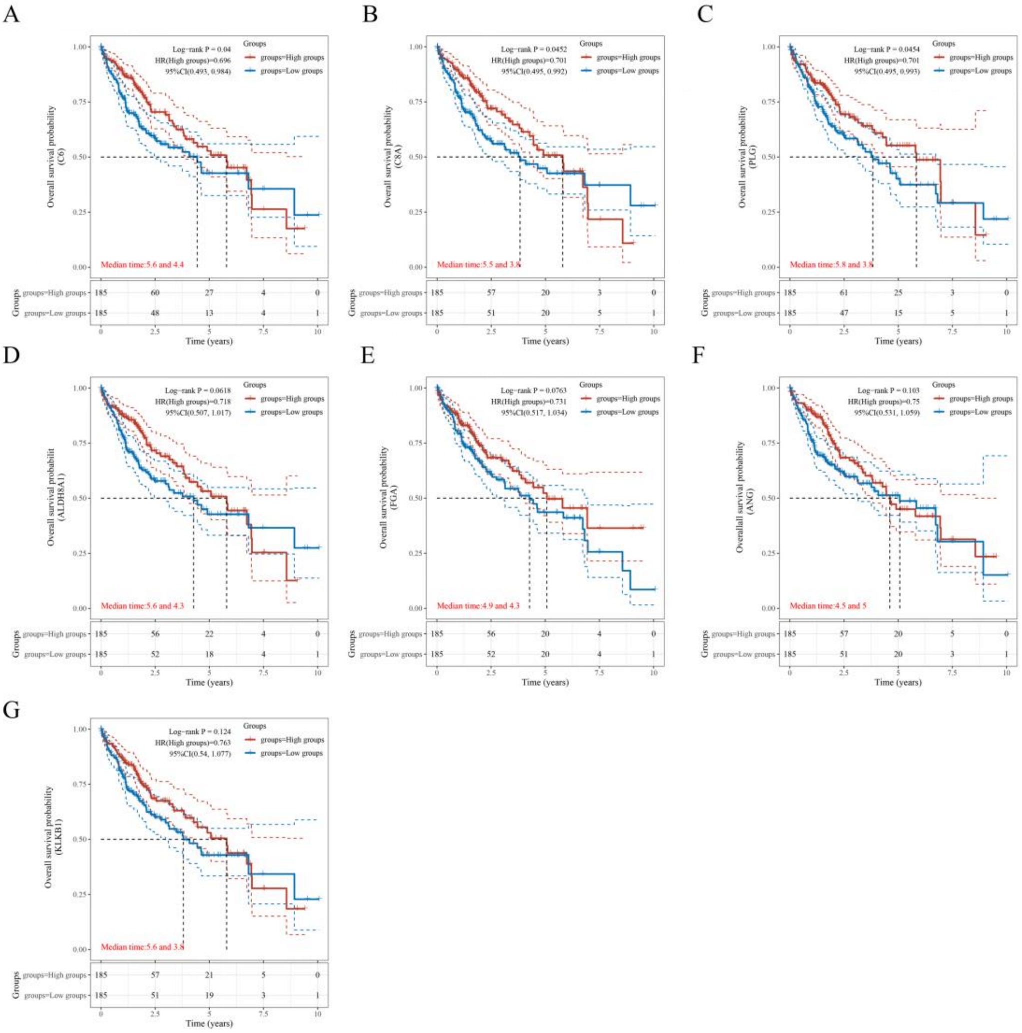

Figure S3 The expression of FTCD in different cell types within liver cancer tissues

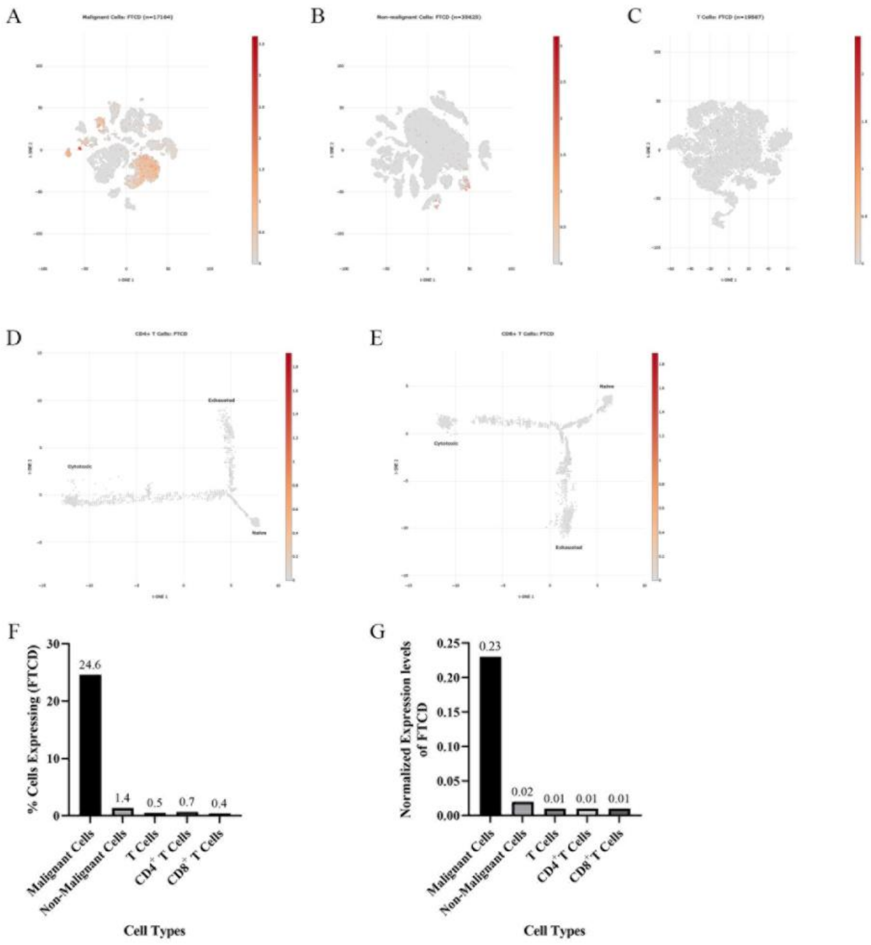

Figure S4 The original image of the exosome detection using western blot. Exosomes were obtained from Hepa1-6 cells (left) and AML12 (right).

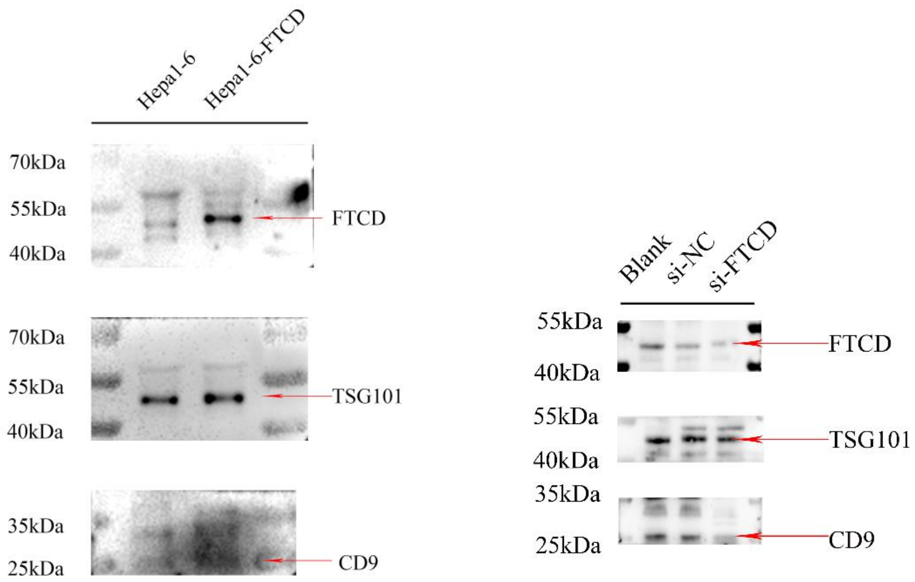

Figure S5 The original image of the detection of the expression of key enzymes in glycolysis progression using western blot.

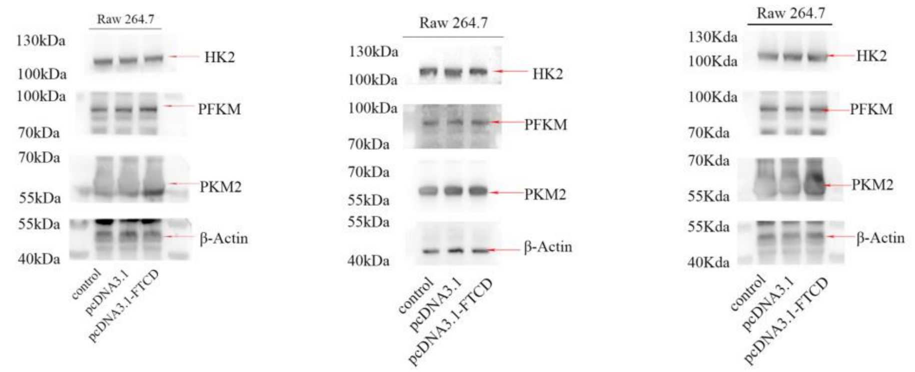

Supplement: Supplementary file 1 [file biomolecules-14-00041-s001.zip › Supplementary Figures.pdf]
