# Supplementary material for: Exosome-Related FTCD Facilitates M1 Macrophage Polarization and Impacts the Prognosis of Hepatocellular Carcinoma
Source: Biomolecules. 2023 Dec 28;14(1):41. doi: 10.3390/biom14010041 (PMC10813691; doi:10.3390/biom14010041)
Supplement: Supplementary file 1 [file biomolecules-14-00041-s001.zip › Table S1 Primers and siRNAs used in the study.pdf]

Table S1 Primers and siRNAs used in the study.

Gene ID		Sequence (5′-3′)
β-actin	Forward	CATCCGTAAAGACCTCTATGCCAAC
	Reverse	ATGGAGCCACCGATCCACA
FTCD	Forward	ACAGCCTCTTGAGAGGAACC
	Reverse	GATGGCATCAATCACCTCCTG
HRG	Forward	CTGCACCACAAGTTCTGTCTC
	Reverse	ACTTGTCCAGGGCTTTACGG
C8B	Forward	GTGGATGGCGTTATGGATCTTG
	Reverse	GTGGAGGAACTTGCTTTGAGTG
si-FTCD-1	Sense Sequence	UUUAUUGCCUCCAAACAGGCA
	Antisense Sequence	CCUGUUUGGAGGCAAUAAAGC
si-FTCD-2	Sense Sequence	GGACAUGACGGAUGAUGUAUU
	Antisense Sequence	UACAUCAUCCGUCAUGUCCUU
si-FTCD-3	Sense Sequence	GACGGAUGAUGUAUUUAAAGA
	Antisense Sequence	UUUAAAUACAUCAUCCGUCAU
