# Supplementary material for: Exosome-Related FTCD Facilitates M1 Macrophage Polarization and Impacts the Prognosis of Hepatocellular Carcinoma
Source: Biomolecules. 2023 Dec 28;14(1):41. doi: 10.3390/biom14010041 (PMC10813691; doi:10.3390/biom14010041)
Supplement: Supplementary file 1 [file biomolecules-14-00041-s001.zip › Table S2 The DEGs shared in GSE36376, GSE55092, and GSE121248 datasets.pdf]

Table S2 The DEGs shared in GSE36376, GSE55092, and GSE121248 datasets

	Gene name
Up-regulated Genes (55)	SPINK1 TKT HSPB1 S100P CCDC34 AKR1B10 SMYD3 COL15A1 RACGAP1 COX7B2 AURKA MCM3 CPD THY1 CCL20 SPP1 SQLE MELK PODXL CDC20 RFC4 PTTG1 TP53I3 RGS5 ASPM TBCE PLCB1 SRXN1 UBE2C NQO1 STXBP6 CCNB2 PRC1 TOP2A FEN1 TXNRD1 CNIH4 PSMD4 DKK1 COL4A1 CAP2 TOMM40L HMMR LYZ GPC3 MCM6 RRAGD LCN2 AKR1C3 PEG10 KIAA0101 CDKN3 NCAPG ACSL4 NUSAP1
Down-regulated Genes (174)	CYP26A1 BBOX1 ACOT12 GSTA1 IGF1 CYP39A1 C1R FAM134B PROZ C8A HRG ZG16 MBL2 RCL1 SLCO1B3 DEFB1 ACSM3 GHR CLEC1B BHMT PON1 STEAP3 SHBG ATOH8 DNASE1L3 PLIN2 BCHE CPEB3 C8orf4 HAO1 ID1 CRHBP ASS1 F9 IGFALS PHLDA1 SLC38A4 DBH MT1M SLC39A5 SRD5A2 EGR1 ECM1 AKR7A3 CYP4V2 FCN2 KLKB1 MT1G CYP2A6 PGLYRP2 SLC22A1 ALDH6A1 APOA5 CHST4 RCAN1 PANK1 PHGDH ARG1 KBTBD11 PCK1 ADH1C DPYS CYP2C9 CYP2A7 CYP2E1 CTH RND3 IL1RAP RDH16 ANG DMGDH TMEM27 AFM CSRNP1 THRSP CYP4A11 AGXT2 MT1X ACAA2 C7 AADAT NNMT CPED1 EPHX2 APOF FAM13A ACSM5 SLC7A2 SDS ETNPPL HPD SLC25A47 SKAP1 SLC10A1 CYP1A2 MT1E ANXA10 TTC36 C8B TAT CYP4F2 FGA FOS ALDH8A1 NAT2 MASP2 AKR1D1 PAMR1 GNMT CXCL12 ACSL1 ACAA1 CA2 FBP1 ADH4 OIT3 GLYAT ADH1A CETP INMT HBB ENO3 LECT2 PLG SOCS2 SLC13A5 SPP2 HAO2 MT1F ADH1B LIPC GSTZ1 LY6E CPS1 CNDP1 FCN3 ACACB GBA3 CLEC4G FXYD1 HPX KMO FOSB SLC27A2 CLRN3 CFHR3 MARCO ADH6 MT1H LCAT TDO2 VIPR1 IGFBP3 PLAC8 HAMP CYP8B1 FTCD CIDEB HGFAC PTH1R MT2A ADGRG7 ZGPAT OGDHL PZP CYR61 HSD17B6 CYP3A4 SLC27A5 GLS2 C6 C9 CDHR2 OAT

Notes:

Abbreviations: DEGs, differentially expressed genes.
