# Supplementary material for: Exosome-Related FTCD Facilitates M1 Macrophage Polarization and Impacts the Prognosis of Hepatocellular Carcinoma
Source: Biomolecules. 2023 Dec 28;14(1):41. doi: 10.3390/biom14010041 (PMC10813691; doi:10.3390/biom14010041)
Supplement: Supplementary file 1 [file biomolecules-14-00041-s001.zip › Table S3 The 83 exosome-related DEGs.pdf]

Table S3 The 83 exosome-related DEGs

Gene name	log2 FC	*P* value
NQO1	1.85	4.86667E-06
RACGAP1	1.59	4.2E-11
S100P	1.56	0.000253958
THY1	1.42	1.35333E-11
ACSL4	2.61	6.10008E-12
AKR1B10	3.35	2.62E-08
AKR1C3	1.60	5.83333E-09
CPD	1.16	2.20018E-12
COL15A1	2.01	2.92667E-18
GPC3	3.73	8.36667E-14
HSPB1	1.30	6.4E-11
LCN2	2.19	0.0000048
LYZ	1.67	1.01335E-05
PLCB1	1.49	3.56667E-12
PODXL	1.34	6.12367E-15
SPP1	2.03	1.07E-07
TXNRD1	1.44	5.86667E-09
TKT	1.57	6.06667E-09
TP53I3	1.48	1.25E-11
HPD	-1.92	0.000323
CXCL12	-2.32	7.56667E-17
PZP	-1.38	8.81253E-17
RND3	-1.93	1.47E-15
ACAA2	-1.17	4.06667E-10
AFM	-2.28	1.43E-07
ADH6	-1.29	1.89333E-07
ALDH6A1	-1.37	0.0000001
ALDH8A1	-1.40	3.73333E-07
AKR1D1	-2.97	2.46E-08
AKR7A3	-1.53	3.15333E-11
ANG	-1.15	3.30013E-06
ARG1	-1.32	0.002156667
ASS1	-1.82	3.12667E-15
BHMT	-1.97	1.45667E-05
CDHR2	-2.01	4.66667E-25
CA2	-2.12	2.22333E-09
SLC39A5	-1.17	5.66688E-12
CETP	-2.06	4.06667E-16
CLRN3	-2.50	1.60763E-14
F9	-1.96	4.83333E-05
C1R	-1.26	2.03333E-08

C6	-2.13	1.72333E-06
C7	-2.25	1.17333E-11
C8A	-1.56	5.73333E-08
C8B	-1.41	6.53333E-06
C9	-3.44	0.00000049
CFHR3	-1.70	2.76667E-06
CTH	-1.51	1.28E-06
CYP4A11	-1.56	4.3E-14
DEFB1	-1.43	0.001016667
DPYS	-1.17	6.66801E-05
ENO3	-1.87	1.54667E-15
EPHX2	-1.31	1.12333E-09
ECM1	-1.65	1.65667E-24
FGA	-1.18	1.88334E-11
FCN2	-2.76	2.27E-23
FTCD	-1.52	1.10333E-09
FBP1	-2.48	2.68E-10
BBOX1	-2.70	1.22515E-13
GSTA1	-1.10	0.001573335
GLYAT	-1.91	3.8E-13
HBB	-1.66	2.26E-08
HPX	-1.59	6.2E-05
HRG	-1.64	0.000986667
HAO2	-2.49	1.48667E-11
IGFBP3	-1.84	5.36667E-17
IGFALS	-1.87	3.5E-27
KLKB1	-1.74	1.23333E-11
KMO	-2.24	3.6E-15
LCAT	-2.51	3.3E-20
MASP2	-1.46	3.29E-08
PON1	-1.32	5.80018E-07
PTH1R	-1.27	3.43333E-20
PGLYRP2	-2.47	5.26667E-10
PCK1	-2.59	0.000228
PHGDH	-1.46	6.86667E-14
PLG	-1.69	2.45483E-09
PROZ	-1.45	1.17334E-12
TMEM27	-2.41	7.13333E-18
SPP2	-2.60	1.15333E-06
SPINK1	3.86	4.46667E-09
SHBG	-1.42	1.5E-15
SLC27A2	-1.53	6.06667E-05

Notes:

Abbreviations: DEGs, differentially expressed genes.
