# Supplementary material for: Exosome-Related FTCD Facilitates M1 Macrophage Polarization and Impacts the Prognosis of Hepatocellular Carcinoma
Source: Biomolecules. 2023 Dec 28;14(1):41. doi: 10.3390/biom14010041 (PMC10813691; doi:10.3390/biom14010041)
Supplement: Supplementary file 1 [file biomolecules-14-00041-s001.zip › Table S5 Hub genes with highest degree of conecticity.pdf]

**Table S5** Hub genes with highest degree of conecticity.

| Rank | Name | Degree |
|---|---|---|
| 1 | FTCD | 19 |
| 2 | FGA | 18 |
| 3 | PLG | 18 |
| 4 | HRG | 18 |
| 5 | C8A | 17 |
| 6 | KLKB1 | 15 |
| 7 | ANG | 15 |
| 8 | C6 | 14 |
| 9 | ALDH8A1 | 14 |
| 10 | C8B | 14 |
